# *Streptococcus salivarius* inhibits immune activation by periodontal disease pathogens

**DOI:** 10.1186/s12903-021-01606-z

**Published:** 2021-05-07

**Authors:** Kyle W. MacDonald, Ryan M. Chanyi, Jean M. Macklaim, Peter A. Cadieux, Gregor Reid, Jeremy P. Burton

**Affiliations:** 1 Department of Microbiology and Immunology, Schulich School of Medicine and Dentistry, University of Western Ontario, London, ON Canada; 2 Canadian Centre for Human Microbiome and Probiotic Research, Lawson Health Research Institute, London, ON Canada; 3 Department of Biochemistry, Schulich School of Medicine and Dentistry, University of Western Ontario, London, ON Canada; 4 School of Health Sciences, Fanshawe College, London, ON Canada; 5 Department of Surgery, Division of Urology, Schulich School of Medicine and Dentistry, University of Western Ontario, London, ON Canada

**Keywords:** *Streptococcus salivarius*, Periodontal disease, Immune inhibition, Probiotics, Chewing gum, *Porphyromonas gingivalis*, *Aggregatibacter actinomycetemcomitans*, *Fusobacterium nucleatum*

## Abstract

**Background:**

Periodontal disease represents a major health concern. The administration of beneficial microbes has been increasing in popularity over efforts to manipulate the microbes using antimicrobial agents. This study determined the ability of *Streptococcus salivarius* to inhibit IL-6 and IL-8 production by gingival fibroblasts when activated by periodontal pathogens and their effect on the salivary microbiome.

**Methods:**

Primary human gingival fibroblasts were challenged with *Porphyromonas gingivalis*, *Aggregatibacter actinomycetemcomitans* and *Fusobacterium nucleatum* and a combination of all three. IL-6 and IL-8 cytokine release were measured. Using this same model, *S. salivarius* K12, M18 and different supernatant and whole-cell lysate fractions of *S. salivarius* K12 were administered to pathogen-induced fibroblasts. A patient study of healthy participants was also conducted to determine the effect *S. salivarius* K12 had on the native microbiome using 16S next generation sequence analysis.

**Results:**

All pathogens tested induced a significant IL-6 and IL-8 response. *S. salivarius* K12 or M18, did not exhibit an increase in inflammatory cytokines. When either of the probiotic strains were co-administered with a pathogen, there were significant reductions in both IL-6 and IL-8 release. This effect was also observed when gingival fibroblasts were pre-treated with either *S. salivarius* K12 or M18 and then stimulated with the oral pathogens. Chewing gum containing *S. salivarius* K12 did not alter the salivary microbiome and did not increase inflammatory markers in the oral cavity.

**Conclusion:**

*S. salivarius* K12 and M18 prevented immune activation induced by periodontal disease pathogens. *S. salivarius* K12 did not alter the salivary microbiome or induce immune activation when administered as a chewing gum. These results warrant further study to determine if it may be an effective treatment in a model of periodontal disease.

**Supplementary Information:**

The online version contains supplementary material available at 10.1186/s12903-021-01606-z.

## Background

Periodontal disease is characterized by inflammation of the tissues that surround and support teeth, including the gingiva and periodontal ligaments. It is believed that up to 50% of North American adults suffer from some form of periodontal disease, making it a major public health concern [1]. The oral cavity is abundant in microbial life, collectively referred to as the “oral microbiota”. During disease, the oral microbiota shifts from a Gram-positive-dominated community to one comprised mainly of Gram-negative bacteria [2]. Bacteria classically considered to be strongly associated with periodontal disease include *Porphyromonas gingivalis*, *Aggregatibacter actinomycetemcomitans* and *Fusobacterium nucleatum* [3]. These are anaerobic bacteria that trigger the release of pro-inflammatory cytokines, leading to immune cell recruitment, tissue destruction, and eventual bone loss. Cytokines important in this destructive cycle include IL-1β (bone resorption, metalloproteinase production), IL-6 (B-cell activation), IL-8 (attraction and activation of neutrophils), and TNF-α (bone resorption) [4].

Oral disease is the fourth most expensive disease to treat worldwide [5] and simple and accessible solutions are needed. Combined with the increase in antibiotic resistance, many novel therapeutic approaches are being developed to aid in oral health and minimizing the impact on the patient. One of these include the use of beneficial bacteria to defend the oral microbiota from a dysbiotic state and exacerbating disease. It is important that a treatment strategy maintains the integrity of the native microbiome without compromising it. Also, a treatment involving live bacteria should remain undetected from the immune system or risk worsening disease symptoms or decreasing treatment efficacy. There are several mechanisms of action by which probiotics exert a beneficial effect either directly or indirectly that may help in the oral cavity. The most useful in terms of protection from oral pathogens is through direct competition with another organism by the production of natural antimicrobial compounds, known as bacteriocins [6]. Other mechanisms enhancing immune regulation [7], improving the integrity of epithelial barriers and function of tight junctions [8, 9], and production of metabolites [10], enzymes, co-factors, and vitamins [11], all of which benefit the health of the host.

Probiotics designed to treat periodontal disease have been tested with promising results. Teughels et al. [16] examined the daily usage of lozenges containing *Lactobacillus reuteri* by patients suffering from chronic periodontitis following standard dental scaling and root planing. This treatment resulted in a significant reduction in pocket depth and attachment gain in deep periodontal pockets, as well as a decrease in *P. gingivalis* compared to those subjects who received a placebo lozenge. A similar study using *Lactobacillus salivarius* WB21-containing tablets demonstrated the ability of this bacterium to reduce the plaque index and periodontal pocket depth in subjects at high risk of periodontal disease [17]. The major metabolic end product of many of these potential probiotics is lactic acid which may have a negative impact on tooth decay over time. For the best result, a non-acid secreting, bacteriocin-producing, colonizer of the oral microbiota may be preferable. *Streptococcus salivarius* is a Gram-positive bacterium that colonizes the human oral cavity throughout the host's life and is generally associated with health [18]. *S. salivarius* K12 and M18 have in vitro inhibitory activity against another oral-pathogen, *Streptococcus pyogenes* [19]. Both strains encode multiple bacteriocins [20, 21], are safe for human consumption [22–24], and can persist in the human oral cavity [25, 26], particularly on the tongue dorsum and other mucosal membranes. *S. salivarius* K12 has been shown in placebo-controlled studies to prevent recurrent streptococcal induced pharyngitis in adults [27] and children [28], as well as reduce halitosis by limiting the production of volatile sulphur compounds from anaerobic bacteria. *S. salivarius* M18 consumption was able to reduce dental plaque scores and the concentration of *S. mutans* in children [29].

As periodontal disease is primarily inflammation driven, and *S. salivarius* K12 and M18 have a proven record of safety and efficient colonization in the human oral cavity, we set out to characterize whether *S. salivarius* K12 or M18 can modulate inflammatory factors produced by human gingival fibroblasts exposed to common dental pathogens and in healthy volunteers, whether changes in the salivary microbiome or secreted cytokines resulted upon increased exposure to *S. salivarius*.

## Methods

### Ethics approval

The study was approved by the Health Sciences Research Ethics Board at The University of Western Ontario (REB 104641, 03/01/2014) and the Clinical Research Impact Committee at the Lawson Health Research Institute (R-13-523). Consent for publication was granted by all participants and any identifying information was removed.

#### Cultures and growth conditions

Strains used in this study are listed in Table 1. *S. salivarius, S. mutans* 25175*, C. albicans* and the nine indicator strains (I1 to I9) were maintained on Brain Heart Infusion medium containing 0.6% (w/v) yeast extract. *A. actinomycetemcomitans*, *P. gingivalis*, and *F. nucleatum* were grown anaerobically on Columbia Blood agar (CBA) containing 5% sheep’s blood at 37 °C in an anaerobic chamber containing 85% (v/v) N_2_, 10% (v/v) H_2_ and 5% (v/v) CO_2_*. Lactobacillus* strains were maintained in De Mann, Rogosa, Sharpe (MRS). When required, 1.5% (w/v) agar was used for propagation on plates.

**Table 1:** Cultures used in this study

Microorganism	Strain
Yeast	
*Candida albicans*	TIMM 1768
Bacteria	
*Aggregatibacter actinomycetemcomitans*	Y4 (ATCC 43718)
*Porphyromonas gingivalis*	ATCC 33277
*Fusobacterium nucleatum*	ATCC10593
*Streptococcus mutans*	ATCC25175
*Streptococcus salivarius*	K12
*Streptococcus salivarius*	M18
*Lactobacillus reuteri*	RC-14
*Lactobacillus plantarum*	Lp-2001
*Lactobacillus helveticus*	LAFTI L-10
Bacterial indicator strains	
*Micrococcus luteus*	I1 Courtesy of J.R. Tagg (Otago)
*Streptococcus pyogenes* M-type 52	I2 Courtesy of J.R. Tagg (Otago)
*Streptococcus constellatus*	I3 Courtesy of J.R. Tagg (Otago)
*Streptococcus uberis*	I4 Courtesy of J.R. Tagg (Otago)
*Streptococcus pyogenes* M-type 4	I5 Courtesy of J.R. Tagg (Otago)
*Lactococcus lactis* ssp. lactis	I6 Courtesy of J.R. Tagg (Otago)
*Streptococcus pyogenes* M-type 87	I8 Courtesy of J.R. Tagg (Otago)
*Streptococcus dysgalactiae*	I9 Courtesy of J.R. Tagg (Otago)

#### Primary human gingival fibroblasts

Gingival fibroblasts were cultured from explanted tissue obtained from healthy volunteers undergoing periodontal procedures in the Oral Surgery Clinic (Western University, Canada) in accordance with the guidelines of the University’s Research Ethics Board (REB 13937E) with informed patient consent. Periodontal fibroblasts were isolated from four patients and were routinely cultured in minimum essential medium (MEM) supplemented with 10% (v/v) fetal bovine serum and 100 mM L-glutamine in a humidified incubator at 37 °C and 5% CO_2_. Experiments were carried out on the gingival fibroblasts between passages 4 to 9. Fibroblasts were inoculated (5 × 10^5^ cells) in a 24-well plate with 500 µl MEM supplemented medium and grown for 48 h to reach confluency.

#### Simultaneous bacterial antagonism

Simultaneous bacterial antagonism assays were conducted as previously described [19]. Briefly, overnight cultures of the indicator strains or pathogens of interest (Table 1) were evenly spread over the surface of a CBA plate. Individual colonies of *S. salivarius* K12 and M18 were used to stab-inoculate the CBA plate and incubated for 48 h at 37 °C in 5% CO_2_. Zones of inhibition surrounding the stab inoculum were used to assess the direct antagonistic effect the bacteria had on pathogen growth.

#### Deferred bacterial antagonism

A single colony of *S. salivarius* K12 or M18 was used to inoculate a 1 cm wide streak on a CBA plate and incubated at 37 °C in 5% CO_2_ for 18 h. The bacterial growth was removed from the plate using a sterile cotton swab then sterilized by chloroform vapour for 20 min. After drying, the indicator strains and pathogens were inoculated onto the plate as a perpendicular line to previous growth. The plate was further incubated for at 37 °C in 5% CO_2_ for 48 h. Since only the secreted by-products from the probiotic bacteria remained, any inhibitory activity to pathogen growth can be attributed to a metabolite secreted during normal growth and not stimulated through direct competition.

#### Co-aggregation to periodontal pathogens

Overnight cultures of each bacterial strain were centrifuged at 3000*g* for 10 min and washed 3 times in sterile PBS. Cultures were resuspended in a final volume of PBS to achieve an optical density (OD_600_) of 1.0. Each pathogen was mixed in equal parts with either *S. salivarius* K12 or M18. The turbidity of the cultures were recorded after 8 h together and compared to the individual culture alone. *C. albicans* and *S. mutans* were used as positive and negative controls, respectively, based on well-known coaggregation abilities. Cultures were given a score based on observed aggregation.

#### *S. salivarius* attachment to primary human gingival fibroblasts

Primary human gingival fibroblasts were processed as described above. Overnight cultures of *S. salivarius* K12 and M18 were centrifuged at 3000×*g* for 10 min and resuspended in the same volume of phosphate buffered saline. This was repeated three times to remove residual bacterial media. *S. salivarius* K12 and M18 were resuspended in supplemented MEM and added to the gingival fibroblasts at a multiplicity of infection (MOI) of 25:1 and incubated for 8 h at 37 °C in 5% CO_2_. The monolayers were washed three times with sterile PBS to remove non-adherent bacteria. Triton X-100 (0.1% v/v) was added to lyse the fibroblasts, releasing adherent *S. salivarius* K12. Bacterial CFUs were determined using dilution plating on CBA. Plates were incubated at 37 °C in 5% CO_2_ overnight**.**

#### Gingival fibroblast challenge and cytokine analysis

Anti-inflammatory effects of *S. salivarius* K12 and M18 were examined using a gingival fibroblast challenge model. Fibroblasts were prepared in 24-well plates as explained above. *S. salivarius*, pathogen or a combination were added to the fibroblasts at a MOI of 25:1. Bacteria were co-incubated with the fibroblasts for 8 h after which the culture supernatant was collected, briefly centrifuged to remove larger debris, and stored at − 20 °C for further analysis. Similarly, to examine the effect of pre-treatment with *S. salivarius* strains, either *S. salivarius* K12 or M18 were applied to gingival fibroblasts 30 min prior to the addition of the periodontal pathogens. This was then further incubated for 8 h, the supernatant collected, briefly centrifuged and stored at − 20 °C until further analysed. In all samples, the concentration of IL-6 and IL-8 were determined using a multiplex immunoassay kit (Luminex).

#### Supernatant analysis

To determine whether *S. salivarius* K12 produced any soluble anti-inflammatory factors, it was grown overnight at 37 °C, centrifuged, and the resultant supernatant was 0.22 µm filter-sterilized, then applied to *F. nucleatum* stimulated fibroblasts for 8 h. For further analysis, *S. salivarius* K12 supernatant was fractionated using a 10 kDa (Centricon® Plus-70), with both the < 10 kDa fraction and > 10 kDa tested on stimulated fibroblasts.

#### Preparation of freeze/thaw extract

To assess the production of intracellular compounds produced by *S. salivarius* K12 that may inhibit immune activation of gingival fibroblasts by *F. nucleatum*, a freeze thaw extract from a bacterial lawn of *S. salivarius* K12 was prepared. A lawn of *S. salivarius* K12 was grown on CBA for 48 h at 37 °C in 5% CO_2_. The plate was placed at − 80 °C for 4 h, thawed at room temperature, and the resulting liquid was collected from the degraded matrix. This was 0.22 µm filter sterilized. Additionally, fractions of this freeze thaw extract were subjected to heat treatment at 80 °C for 10 min or digested for 10 min using 0.05% trypsin at 37 °C with 5% CO_2_. These fractions were added to *F. nucleatum* and were co-incubated to stimulate fibroblasts for 8 h and IL-8 was measured as described above.

#### Probiotic gum study design

Participants were recruited between the ages of 20–60 years with general good oral health. Participants were excluded if they had any oral disease, an oral implanted device, were currently taking antibiotics, or had a dental appointment scheduled during the course of the study. A total of twenty healthy adult volunteers were selected and assigned to two study groups (n = 10), matched for age and sex. Participants received either chewing gum containing *S. salivarius* K12 (CulturedCare™ with BLIS K12™; Group 1) or regular gum tablets lacking *S. salivarius* K12 (Group 2). Each individual was assigned a unique identifier code, to ensure anonymity and that we would be blinded to which group a sample belonged too. Both gum types were similar in taste, appearance, and texture. Participants were supplied enough gum tablets to last the duration of the study. Additional file 1: Figure S1 demonstrates the overview of the study design. A 3 mL sample of unstimulated saliva was collected at appropriate time points for 7 days followed by a further 7 day wash out period. Samples were stored at − 80 °C until all samples were received from all participants. One participant was not able to provide all samples and was excluded from analysis.

#### In vivo cytokine release

IL-1β, IL-6, IL-8, and TNF-α were measured in saliva samples using multiplexed immunoassay as described above according to the manufacturer’s instructions (Bio-Rad Laboratories Inc., Hercules, CA). A Bio-Plex 200 readout system was used (Bio-Rad), that utilizes Luminex® xMAP fluorescent bead-based technology (Luminex Corporation, Austin, TX).

#### Salivary microbiome analysis

DNA was extracted from saliva samples using the DNeasy PowerSoil 96-well Isolation Kit (Qiagen). The extraction was carried out as per the manufacturers protocol, with two changes; the addition of a 10 min incubation step at 65 °C in a bead bath prior to the bead-beating step, and the centrifugation times for each step were doubled. In total, 500 μl of saliva was used for the extraction of 94 samples. Extracted samples were amplified by PCR for the V4 region of the 16S rRNA gene using barcoded primers as follows: V4L (forward) 5’ GTGCCAGC[CA]GCCGCGGTAA 3’ and V4R (reverse) 5’ GGACTAC[ATC][ACG]GGGT[AT]TCTAAT 3’. Amplification was carried out in a 42 µL reaction with 10 µL of each primer (3.2 pMol/µL stock), 20 µL GoTaq hot start master mix (Promega) and 2 µL extracted DNA. Thermocycling conditions were as follows: initial hot start activation at 95 °C for 2 min, then 25 cycles of 1 min at 95 °C for denaturation, 1 min 55 °C for primer annealing, and 1 min at 72 °C for extension. PCR products were quantified with a Qubit 2.0 fluorimeter and high sensitivity dsDNA specific fluorescent probes (Life Technologies). Samples were mixed at equimolar concentrations and purified with the QIAquick PCR Purification kit (Qiagen). The pooled product was sent to the London Regional Genomics Centre (Robarts Research Institute, Western University, London, Canada) for sequencing on the Illumina MiSeq platform using the 600-cycle kit to produce 2 × 300 paired-end reads. Using in-house Perl and Shell scripts, reads were retained if sequence matched the primer while allowing 2 bp mismatches, and with perfect matches to expected sequence barcodes. Paired reads passing this filter were overlapped using pandaseq (https://github.com/neufeld/pandaseq) to produce full-length V4 sequences assigned by sample. Operational taxonomic units (OTUs) were constructed by clustering V4 reads at 97% sequence identity using USearch v. 7 (http://www.drive5.com/usearch/). OTUs were retained if they represented at least 0.1% relative abundance of any one sample. The most abundant sequence in the cluster was used as the reference sequence for taxonomic classification. The reference OTU sequences were compared to the ribosomal database project v11.2 (RDP; https://rdp.cme.msu.edu) using Seqmatch v.3, and the lowest common taxonomy was retained out of the top 20 hits with an S_ab score ≥ 0.5. OTU sequences from differential taxonomic groups were further validated by BLAST against the Human Oral Microbiome Database (HOMD) v. 13.2 (http://www.homd.org). The OTU table with assigned taxonomies was imported into QIIME (http://qiime.org) for exploratory analyses including summarizing reads to different taxonomic levels, generating beta diversity with weighted UniFrac distance based on OTU sequence alignment with MUSCLE, and principal coordinate analysis (PCoA). Bar, stripchart, and PCoA plots were generated using R.

#### Statistical analysis

Experiments were minimally performed in triplicate. Data was analysed using GraphPad Prism (Version 9.0.0 for Windows, GraphPad Software, San Diego, California, USA) using a one- or two-way ANOVA where appropriate with either the Dunnett’s or Tukey’s post-hoc multiple comparison test as described in the figure legend. Dunnett’s post-hoc test was chosen when comparing multiple groups to a control group. Tukey’s post-hoc test was chosen when comparing multiple groups to each other. For microbiome analysis, between-group comparisons for differential microbiota analyses were conducted with ALDEx2 package (http://www.bioconductor.org/packages/release/bioc/html/ALDEx2.html) in R. Taxonomic clusters were considered differential between groups with an adjusted *p*-value < 0.01 using Welch’s t-test with Benjamini–Hochberg multiple test correction, and with an effect size ≥ 1.5.

## Results

### *S. salivarius* interaction with oral pathogens

Based on previous literature, it was confirmed that both *S. salivarius* K12 and M18 showed strong direct- and deferred-inhibition to all 9 indicator strains (Table 2), giving it P-Type 7–7-7. Upon an extended spectrum analysis, neither strain K12 nor M18 were able to inhibit *C. albicans*, *S. mutans*, *P. gingivalis*, *F. nucleatum*, nor *A. actinomycetemcomitans* via direct- and deferred-antagonism assays (Table 2). A coaggregation assay demonstrated that both K12 and M18 were able to co-aggregate moderately with *P. gingivalis* and *F. nucleatum* and weakly with *A. actinomycetemcomitans* (Table 3). No co-aggregation was observed with either strain and *S. mutans* (Table 3).

**Table 2:** Simultaneous and deferred bacterial antagonism

Indicator Strain	Simultaneous antagonism *S. salivarius* producer strain		Deferred antagonism *S. salivarius* producer strain	
	K12^†^	M18^‡^	K12	M18
*Porphyromonas gingivalis* ATCC 33277	−^§^	−	−	−
*Fusobacterium nucleatum* ATCC10593	−	−	−	−
*Aggregatibacter actinomycetemcomitans* Y4	−	−	−	−
*Streptococcus mutans* ATCC25175	−	−	−	−
*Candida albicans* TIMM 1768	−	−	−	−
*Micrococcus luteus* (I1)	++^¶^	++	++	++
*Streptococcus pyogenes M-type 52* (I2)	++	++	++	++
*Streptococcus constellatus* (I3)	++	++	++	++
*Streptococcus uberis* (I4)	++	++	++	++
*Streptococcus pyogenes M-type 4* (I5)	++	++	++	++
*Lactococcus lactis ssp. lactis* (I6)	++	++	++	++
*Streptococcus pyogenes M-type 28* (I7)	++	++	++	++
*Streptococcus pyogenes M-type 87* (I8)	++	++	++	++
*Streptococcus dysgalactiae* (I9)	++	++	++	++

Inhibition of bacterial growth by *S. salivarius* K12 or M18. Results were consistent across the three experiments conducted

^†^*Streptococcus salivarius* K12; ^‡^*Streptococcus salivarius* M18; ^§^(−) No inhibition; ^¶^(++) Strong inhibition

**Table 3:** Bacterial co-aggregation

*S. salivarius *strain	Pathogen			
	*P. gingivalis*	*F. nucleatum*	*A. actinomycetemcomitans*	*S. mutans*
K12^†^	++^§^	++	+	−^¶^
M18^‡^	++	++	+	−

Ability of *S. salivarius* K12/M18 to co-aggregate in solution with various pathogens. Results were consistent across the three experiments conducted

^†^*S. salivarius* K12; ^‡^*S. salivarius* M18; ^§^(++) Moderate precipitation with evenly turbid supernatant and evidence of flocculation; ^¶^(−) No co-aggregation, evenly turbid suspension

### Primary human gingival fibroblast challenge

**Fig. 1:**
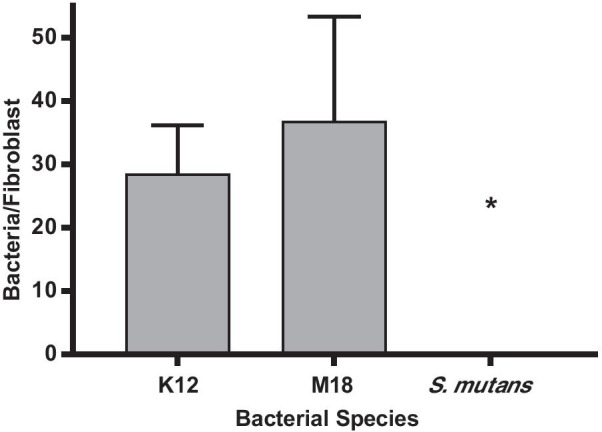
Bacterial adherence to human gingival fibroblasts. Bacterial attachment to primary human gingival fibroblasts in vitro following 8 h co-incubation. *S. salivarius* K12 (K12); *S. salivarius* M18 (M18); *S. mutans* ATCC25175. Assay was carried out in triplet on three separate occasions. Samples were analysed using a one-way ANOVA with Dunnett’s multiple comparison test with K12 as the control (*p < 0.05 compared to K12 attachment). Error bars represent ± standard error of the mean

Both *S. salivarius* K12 and M18 were able to adhere to the primary human gingival fibroblasts at a ratio of 30 bacterial cells per fibroblast with no appreciable difference observed between them (Fig. 1). As a comparison, a widely used probiotic *L. reuteri* RC-14, adhered at a ratio of 5 bacterial cells per fibroblast (Additional file 2: Fig. S2). Therefore, it was sought to determine if the ability of K12 and M18 to co-aggregate with oral pathogens would negatively impact the inflammatory response of the oral cavity.

**Fig. 2:**
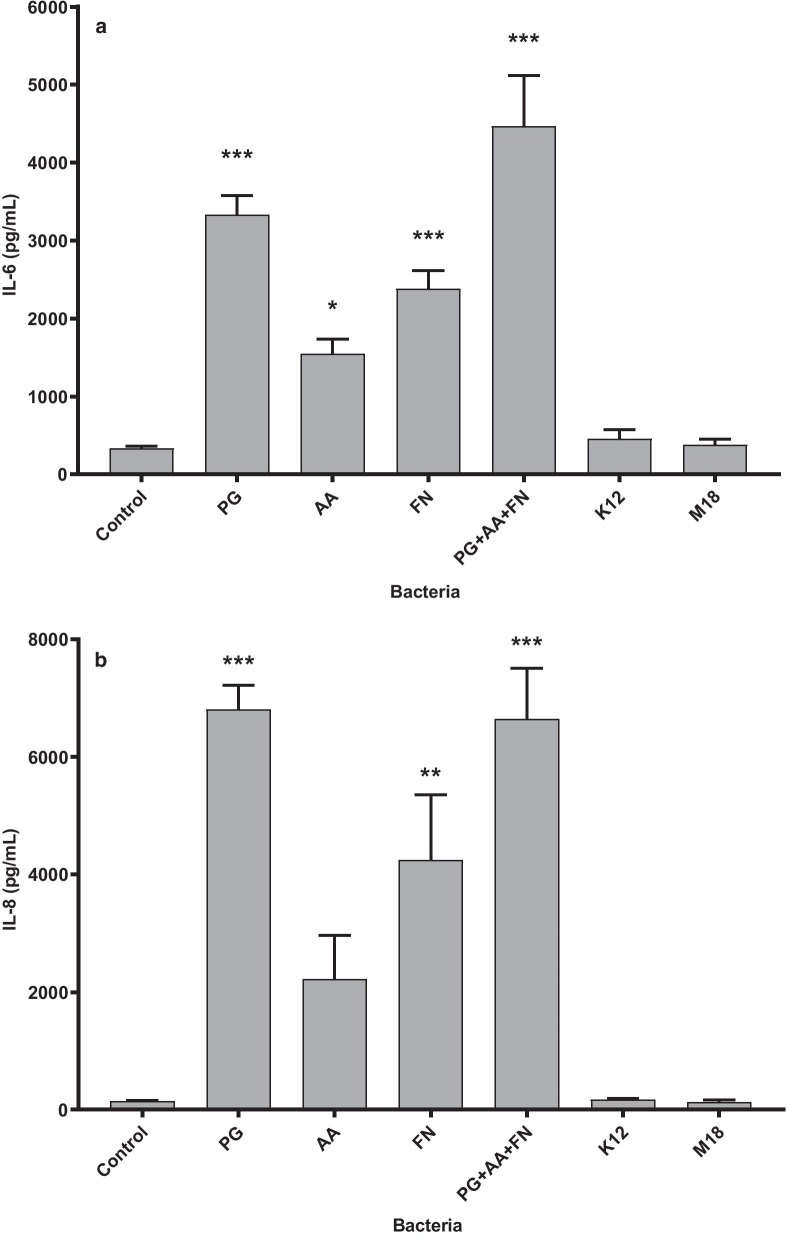
Stimulation of cytokine production from human gingival fibroblasts. IL-6 (**a**) and IL-8 (**b**) release by primary human gingival fibroblasts induced by 8-h co-incubation with *P. gingivalis* (PG); *A. actinomycetemcomitans* (AA); *F. nucleatum* (FN); *S. salivarius* K12 (K12); or *S. salivarius* M18 (M18). Samples were analysed using a one-way ANOVA with Dunnett’s multiple comparison test as compared to control (*p < 0.05, **p < 0.01, ***p < 0.001 compared to control). Error bars represent ± standard error of the mean

Baseline IL-6 and IL-8 concentrations from unstimulated gingival fibroblasts were 330.3 pg/ml and 149.1 pg/ml, respectively (Fig. 2). All three periodontal pathogens tested significantly increased the release of IL-6 (Fig. 2a) and IL-8 (Fig. 2b). *P. gingivalis* significantly activated the greatest IL-6 (3333.9 pg/ml; *p* < 0.001) and IL-8 release (6812.5 pg/ml; *p* < 0.001) from gingival fibroblasts, followed by *F. nucleatum* (IL-6 = 2382.4 pg/ml, *p* = 0.0009; IL-8 = 4251.8 pg/ml; *p* = 0.005) and lastly, *A. actinomycetemcomitans* (IL-6 = 1551 pg/ml; NS at *p* = 0.0595; IL-8 = 2225.3 pg/ml, NS at *p* = 0.278). The combination of the three pathogens further stimulated IL-6 release to 4466.6 pg/ml, greater than any individual strain alone (Fig. 2a), significantly higher than *A. actinomycetemcomitans* alone (*p* < 0.0001) and *F. nucleatum* alone (*p* = 0.0015). The combination did not further increase IL-8 release (6645.7 pg/ml) beyond that of *P. gingivalis* alone (Fig. 2b). Despite the ability to adhere to the fibroblasts, *S. salivarius* K12 or M18 did not induce an IL-6 or an IL-8 response (Fig. 2a, b; NS from control).

**Fig. 3:**
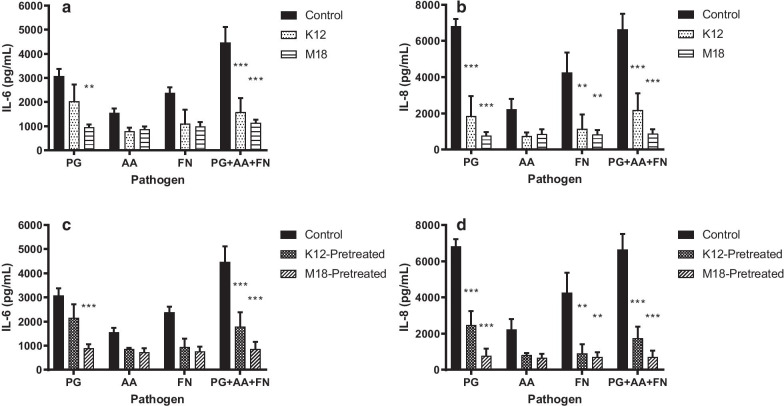
*S. salivarius* reduction of pathogen stimulated cytokine release. IL-6 (**a**) and IL-8 (**b**) release from primary human gingival fibroblasts induced by *P. gingivalis* (PG); *A. actinomycetemcomitans* (AA); *F. nucleatum* (FN) when co-incubated with *S. salivarius* K12 (K12) or M18 (M18). IL-6 (**c**) and IL-8 (**d**) release from gingival fibroblasts induced by the oral pathogens when *S. salivarius* K12 (K12-Pretreated) or M18 (M18-Pretreated) were added as a pre-treatment to the fibroblasts 30 min prior to pathogen challenge. Samples were analysed using a two-way ANOVA with Tukey’s multiple comparison test within each pathogen group (**p < 0.01, ***p < 0.001). Error bars represent ± standard error of the mean

Using the same in vitro model system, *S. salivarius* K12 or M18 were applied either simultaneously to the fibroblasts with *P. gingivalis*, *A. actinomycetemcomitans*, *F. nucleatum* (Fig. 3a, b) or were preincubated with the gingival fibroblasts 30 min prior to being challenged by the pathogens (Fig. 3c, d). When added simultaneously, *S. salivarius* M18 was able to significantly decrease IL-6 production induced by *P. gingivalis* (Fig. 3a; *p* = 0.0012). Both *S. salivarius* K12 and M18 were able to significantly decrease the production of IL-6 induced by the combination of all three pathogens (Fig. 3a; *p* < 0.0001). Similarly, both *S. salivarius* strains K12 and M18 inhibited the production of IL-8 (Fig. 3b) induced by *P. gingivalis* (*p* < 0.001), *F. nucleatum* (*p* = 0.0059, *p* = 0.0021, respectively), and the combination of the three pathogens (*p* < 0.001). Although not all decreases were significant, coadministration of either *S. salivarius* K12 or M18 with any of the pathogens demonstrated a minimum inhibition of 34.1% for IL-6 and 61.5% for IL-8 (Additional file 3: Table S1).

Furthermore, when added as a pre-treatment, *S. salivarius* M18 was able to significantly decrease IL-6 secretion induced by *P. gingivalis* alone (Fig. 3c; *p* = 0.0012). Both *S. salivarius* K12 and M18 were able to significantly inhibit IL-6 induction by the combination of all three pathogens by 60.0% and 80.7%, respectively (Fig. 3c; *p* < 0.001; Additional file 3: Table S1). Similarly, both K12 and M18 were able to significantly inhibit IL-8 induced by *P. gingivalis* (Fig. 3d; *p* < 0.001) and *F. nucleatum* (*p* = 0.0027, *p* = 0.0013) and the combination of all three pathogens (*p* < 0.001). In all samples tested, *S. salivarius* K12 and M18 inhibited cytokine release by gingival fibroblasts with a minimum inhibition of 30% and 63.2% for IL-6 and IL-8, respectively. Importantly, under no circumstances did the addition of the *S. salivarius* K12 or M18 increase the production of IL-6 or IL-8 from pathogen stimulated fibroblasts.

### Determination of anti-inflammatory factor

**Fig. 4:**
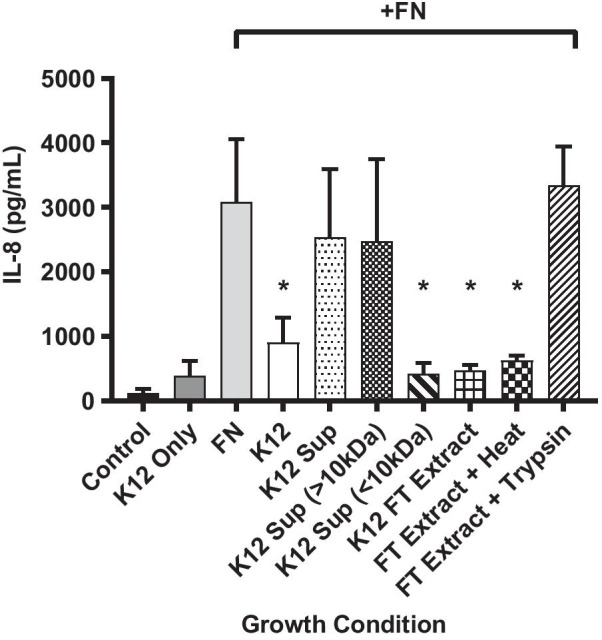
Mechanism of *S. salivarius* K12 mediated IL-8 reduction. IL-8 release by primary human gingival fibroblasts induced by *F. nucleatum* (FN). *S. salivarius* K12 (K12) and various culture supernatants co-administered with FN; Sterile filtered supernatant (K12 Sup); K12 Supernatant fraction > 10 kDa (K12 Sup > 10 kDa); K12 Supernatant fraction < 10 kDa (K12 Sup < 10 kDa); K12 cells Freeze/Thaw Extract (FT); FT Extract heat inactivated at 80 °C 10 min (FT Extract + Heat); FT Extract digested with Trypsin for 10 min (FT Extract + Trypsin). Samples were analysed using a one-way ANOVA with Dunnett’s multiple comparison test compared with control (*p < 0.05). Error bars represent ± standard error of the mean

It was determined prior that neither *S. salivarius* K12 nor M18 were able to directly or indirectly (deferred antagonism) inhibit pathogen growth, and therefore, another mechanism of action must exist to account for the reduction in IL-6 and IL-8 response of gingival fibroblasts induced by these pathogens. Focussing on IL-8 release induced by *F. nucleatum*, different *S. salivarius* K12 fractions were examined to elucidate the factor responsible. Figure 4 demonstrates that, as expected, IL-8 release was inhibited by *S. salivarius* K12 when induced by *F. nucleatum*. Surprisingly, *S. salivarius* K12 filter sterilized whole bacterial supernatant did not prevent IL-8 release; however, when the supernatant was fractionated, the smaller size fraction containing < 10 kDa metabolites was able to significantly inhibit IL-8 release (*p* = 0.0111). This is most likely due to a concentration of the causative agent during the fractionation as to why whole supernatant did not have the same effect. To determine if *S. salivarius* produces the compound when on a solid surface, it was grown on agar plates and crudely lysed through a freeze/thaw process that is known to release the intracellular components into a concentrated fraction that can be easily isolated. The freeze/thaw fraction (FT Extract) significantly inhibited IL-8 release (Fig. 4; *p* = 0.0126). It was also confirmed the agent is heat stable and still active after being treated at 80 °C for 10 min (*p* = 0.0186); however, it was inactivated when subjected to a trypsin digest. Therefore, it can be concluded that the agent responsible is a small molecule, less than 10 kDa in size, heat stable and proteinaceous in nature, secreted in small amounts but not anti-microbial to the pathogens tested based on direct and deferred antagonism assays (Table 2).

### Effects of probiotic gum on the healthy salivary microbiome

To examine the effect a probiotic gum containing *S. salivarius* K12 would have on a healthy microbiome as well as inflammatory markers, a human study consisting of nineteen healthy volunteers was conducted. The treatment regimen is described in Additional file 1: Figure S1. Samples were collected at baseline, 4 h, 24 h, 7 days and 14 days.

**Fig. 5:**
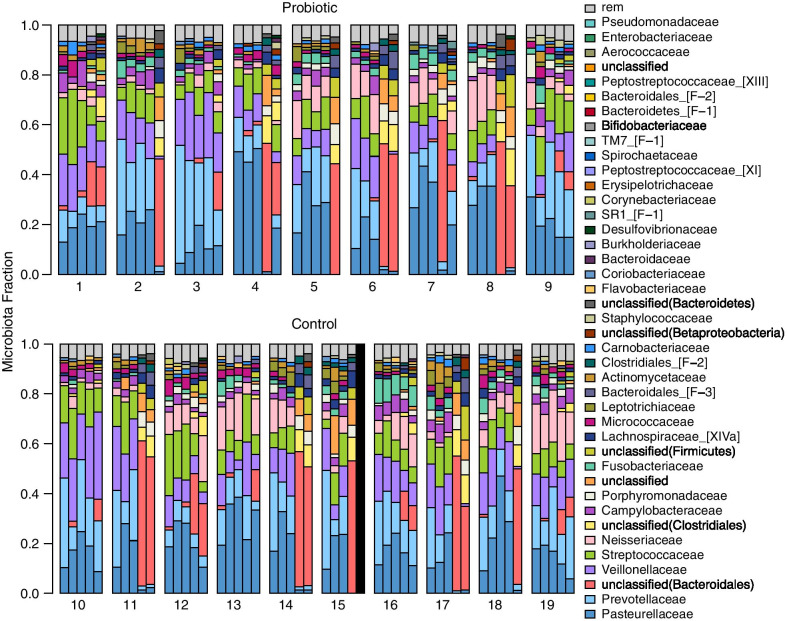
Salivary microbiota profiles. Bacterial composition of the salivary microbiota was assessed in all volunteers using V4 16S rRNA gene sequencing at five time-points; baseline, 4 h, 24 h, 7 days and 14 days. Volunteers were given chewing gum tablets containing either *S. salivarius* K12 (Top Panel) or non-probiotic control gum (Bottom Panel). Each cluster of bars is a single volunteer identified by a subject ID number, and each bar in a cluster is an individual saliva sample ordered (left to right) as; baseline, 4 h, 24 h, 7 days, and 14 days. The black bar indicates an uncollected sample. Colours correspond to the proportion of assigned taxonomic family (listed in the legend on the right), ordered according to total relative abundance from bottom of the plot to top. Groups of sequences that are less than 0.05% relative abundance across all samples, or less than 1% of sequences in an individual are grouped as “rem”. Unclassified families are labelled by their lowest classified taxonomic rank

**Fig. 6:**
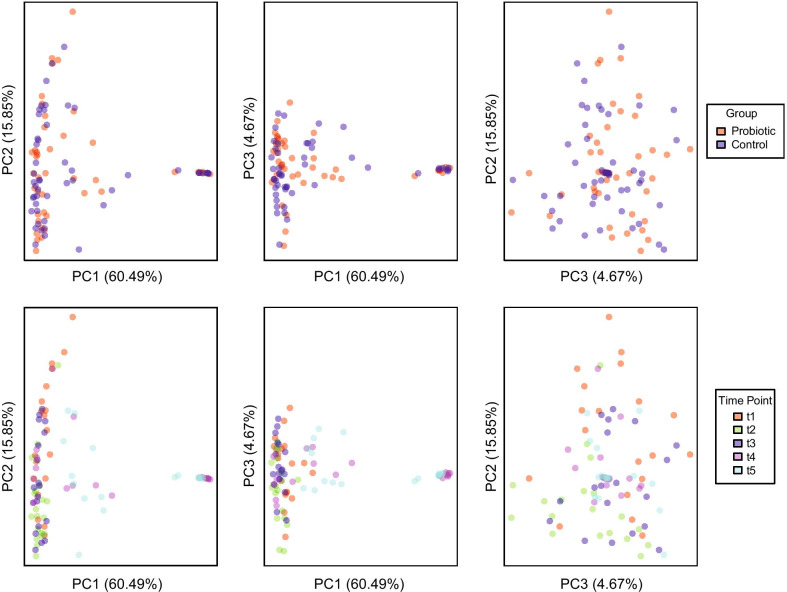
PCoA plots for all saliva samples based on weighted UniFrac distance. Two dimensional PCoA plots representing the first three components of variation between all saliva samples in the dataset. The first component in this analysis represents the most variation explained in the data (in this case 60.49%), with subsequent components representing the next largest variance in the data. Distances between points on the plot represent how similar samples are in terms of microbiota composition and relative abundance. Points on the plot that are closer in space are therefore more similar in their taxonomic distribution. The top and bottom row plots are identical, differentially coloured based on variable of interest (top—study group; bottom—sample collection timepoint)

**Fig. 7:**
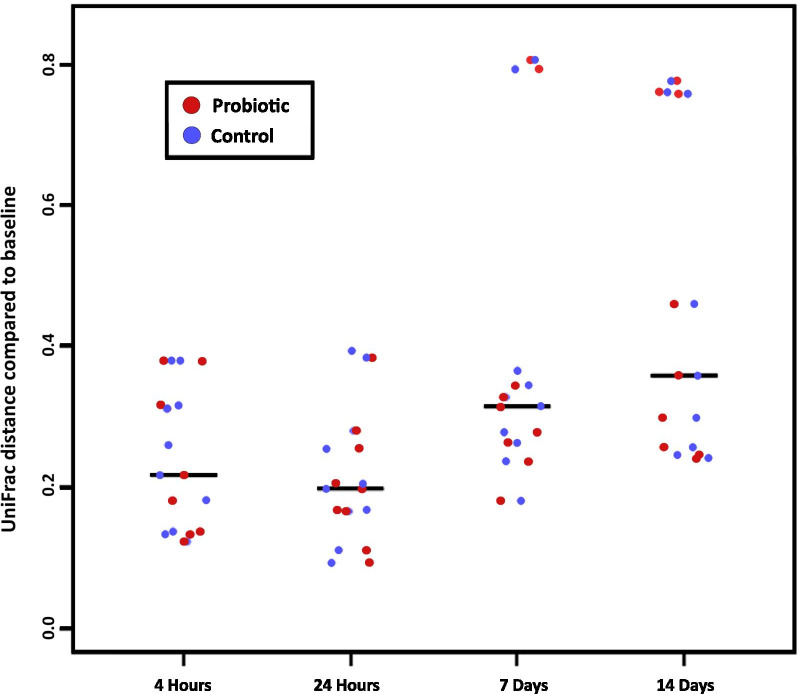
Change in β-diversity measured by weighted UniFrac over time. Weighted UniFrac distance of each saliva microbiota sample compared to that individual's baseline sample at 4 h, 24 h, 7 days, and 14 days. A value of 0 would represent identical microbiota composition between samples, with a value of 1 representing maximal microbiota differences. Sample points are coloured by study group (probiotic—red; control—blue). Lines represent the median UniFrac distance of a given timepoint. Microbiota compositions change over time (regardless of study group), with a subset of individuals changing drastically at 7 and 14 days

Saliva samples were subjected to 16S rRNA gene sequencing via the Illumina MiSeq platform. After filtering and clustering sequenced reads at 97% identity, there were 476 OTUs with > 0.01% total relative abundance in the saliva samples collected from test subjects. A total number of 2,774,309 sequenced reads were included for analysis, with a median of 29,779 ± 13,630 reads per sample. These OTUs were further clustered by taxonomic lineage into 38 family groups of at least 0.05% relative abundance across all samples. Figure 5 shows the most abundant taxonomic families detected at > 10% of total classified reads were: *Porphyromonadaceae* (17.95%), *Pasteurellaceae* (15.97%), *Prevotellaceae* (15.85%), and *Veillonellaceae* (11.22%). Using PCoA (Fig. 6), the samples did not separate by group (probiotic versus control) in examination of the first 3 components (81.01% of the total variance in the data). However, there is a distinct shift in the first component over time with many of the 7-day and 14-day samples differentiating from the earlier timepoints (Fig. 6, bottom row). This differentiation was also independent of group (probiotic versus control). To further examine the apparent time-dependent change in microbiota, the weighted UniFrac distance of all timepoints from individuals in both treatment groups compared to their baseline sample before treatment were plotted (Fig. 7). The median weighted UniFrac distance increased over treatment time indicating a shift in the microbiota. Notably, a subset of the samples at 7 days and 14 days were very distinct from the others.

In order to test if there were any differential taxonomic abundances between groups, a compositional data analysis framework was required and the ALDEx2 toolset was employed to test for significant taxonomic difference between groups at the family level. There were no differences (Benjamini–Hochberg adjusted *p* > 0.01) between the probiotic and control groups at baseline or study endpoint (14 days), nor between these groups at any of the other sample collection timepoints. Therefore, the treatment groups were pooled to test for differences at end of study (14 days) compared to baseline. There were four family-level taxonomic groups with a relative increase in relative abundance (Table 4), and three with a relative decrease in relative abundance (Benjamini–Hochberg adjusted *p* < 0.01 and effect size ≥ 1.5). Examination of the OTUs in the family groups by BLAST to the HOMDB revealed that most of the OTU sequences in *Erysipelotrichaceae* were similar (> 80% sequence identity) to *Erysipelothrix tonsillarum* (HOT_484) or *Solobacterium moorei* (HOT_678).

**Table 4:** Taxonomic groups with significant changes in relative abundance

Family-level taxonomic group	Wt-BH^†^	Effect size^‡^
Relative increase		
Firmicutes; Erysipelotrichia; Erysipelotrichales; Erysipelotrichaceae	1.85E−08	2.04
Bacteroidetes; Bacteroidia; Bacteroidales; Porphyromonadaceae2	4.48E−08	1.87
Bacteroidetes; Bacteroidia; Bacteroidales; Bacteroidaceae	5.25E−06	1.57
Bacteroidetes; Bacteroidia; Bacteroidales; Porphyromonadaceae	6.86E−07	1.51
Relative decrease		
Fusobacteria; Fusobacteria; Fusobacteriales; Leptotrichiaceae	1.39E−05	− 1.77
Actinobacteria; Actinobacteria; Actinomycetales; Actinomycetaceae	1.74E−05	− 1.61
Bacteroidetes; Bacteroidia; Bacteroidales; Prevotellaceae	1.34E−05	− 1.51

^†^Corrected p-value from a paired Welch’s t-test using Benjamini–Hochberg procedure [33]

^‡^The median effect size as estimated by ALDEx2

### Changes in pro-inflammatory cytokine levels

**Fig. 8:**
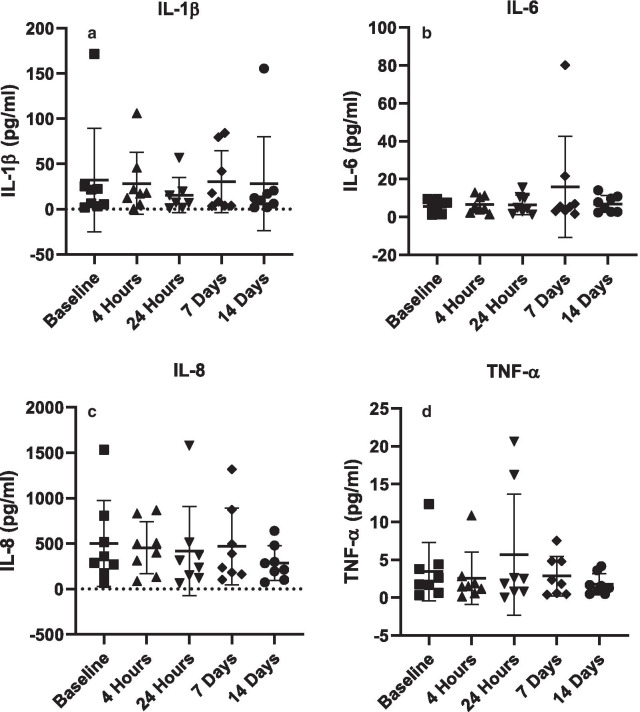
Salivary levels of pro-inflammatory cytokines of volunteers chewing probiotic tablets. Concentration (pg/mL) of IL-1β (**a**); IL-6 (**b**); IL-8 (**c**); TNF-α (**d**) in saliva samples collected from healthy volunteers chewing the probiotic gum at baseline, 4 h, 24 h, 7 days and 14 days. Cytokine levels for each sample were determined individually with the mean concentration for all individuals shown. Samples were analysed using a one-way repeated-measures ANOVA with Tukey’s multiple comparison test. No significant differences were observed. Error bars represent ± standard error of the mean

Concentrations of four pro-inflammatory cytokines (IL-1β, IL-6, IL-8 and TNF-α) linked with periodontal disease were measured in the collected saliva of nine subjects in the probiotic gum group at each timepoint. These were all healthy individuals with no overt oral disease; however, each participant had some degree of these inflammatory cytokines present in their saliva (Fig. 8). None of the cytokines tested at any of the time points were significantly different from the baseline control (NS from baseline). On average, there was 27.27 pg/ml IL-1β, 8.32 pg/ml IL-6, 426.72 pg/ml IL-8, and 3.27 pg/ml TNF-α. To remove participant variation, each subject was individually analysed, but there were no statistically significant differences in cytokine profile observed (Data not shown).

## Discussion

The ability for *S. salivarius* K12 and M18 to directly inhibit the growth of oral pathogens is well documented. The most notable mechanism of action has been attributed to megaplasmids pSsal-K12 and pSsal-M18 encoded by *S. salivarius* K12 and M18, respectively. These megaplasmids encode for many bacteriocins like salivaricin A2 and salivaricin B [20]. Interestingly, neither *S. salivarius* K12 nor M18 were able to directly inhibit the growth of the oral pathogens tested through direct- and deferred-antagonism assays, indicating the inhibition of immune activation was not linked to bacteriocin killing target organisms. This study demonstrated that *S. salivarius* K12 and M18 could interact with other microbes known to cause disease in the oral cavity and influence pathogen-stimulated production of inflammatory mediators from primary human gingival fibroblasts.

It was shown that *S. salivarius* K12 and M18 were both able to adhere to gingival fibroblasts better than another well-known probiotic bacterium, *L reuteri* RC-14. It is possible that this adherence may compete with oral pathogens to locate suitable binding-sites. Manning et al. [30] demonstrated that *S. salivarius* K12 and M18 were able to prevent pneumococcal adherence to pharyngeal epithelial cells through direct competition for pneumococcal binding sites. Interestingly, both *S. salivarius* K12 and M18 were able to directly inhibit pneumococcal growth on solid media but this mechanism was not required to prevent pneumococcal adherence. The current study did not determine pathogen binding to the gingival fibroblasts and if *S. salivarius* K12 or M18 prevented this from occurring. As the active compound was secreted into the supernatant and rendered ineffective upon trypsin treatment, it can be surmised this would not have been the major mechanism of action.

Many species belonging to *Streptococcus* have documented anti-inflammatory properties. This study demonstrated that both *S. salivarius* K12 and M18 were unable to elicit an IL-6 or an IL-8 response from primary human gingival fibroblasts despite being able to adhere to this tissue well. Moreover, both strains were able to inhibit the IL-6 and IL-8 release induced by three oral pathogens, *P. gingivalis*, *A. actinomycetemcomitans*, and *F. nucleatum* individually or used in combination. This inhibition occurred whether that strains were co-administered with the pathogens or supplied prior to pathogen challenge. The causative agent was further investigated and was identified to be a small molecule, < 10 kDa in size, heat stable and proteinaceous. This extract was able to inhibit IL-8 release induced by *F. nucleatum* similar to that of adding *S. salivarius* K12 simultaneously. Therefore, it is believed that the mechanism of action does not target the bacterium to inhibit pathogenesis but must target the host cells to maintain immune homeostasis. These results correlate well with previous studies showing the anti-inflammatory effect of other *S. salivarius* and *S. vestibularis* strains. Kaci et al*.* [31, 32] have demonstrated that strains of *S. salivarius* inhibited TNF-α activation of the NFκ-B inflammatory response of stimulated intestinal epithelial cells, and intra-gastric administration of a live *S. salivarius* significantly inhibited inflammation in mouse models of moderate and severe colitis [31]. The inhibition of NFκ-B activation was also observed using culture supernatants of *S. salivarius* and *S. vestibularis* in an NFκ-B reporter system in the HT-29 cell line [32]. It was determined a small molecule < 3 kDa in size was responsible for the inhibition of TNF-α, IL-1β and IL-8 [32]. It is likely the effector identified in the current study was similar to the one isolated by Kaci et al*.* [32]. Our study focused on *S. salivarius*; however, others have also demonstrated similar decreases in IL-8 release by *S. mitis* and *S. sanguinis* [33, 34] indicating this may be a global mechanism associated with many species of beneficial *Streptococcus.*

Due to the broad range of anti-microbial activity combined with anti-inflammatory properties, and its GRAS-status, probiotic formulations containing *S. salivarius* for different applications are becoming more widespread. For many years, the simple presence or increased relative abundance of certain bacterial species was believed to be the driving force behind many oral diseases. *Streptococcus mutans* was long presumed to be the primary etiological agent of dental caries [35]. In other studies, *P. gingivalis*, *Tannerella forsythia* and *Treponema denticola* (categorized together as the "red complex") were reported to be closely linked with periodontal disease [36]. However, studies using high throughput sequencing techniques have shown that these assumptions are over-simplistic, with diseases often being polymicrobial in nature [37, 38], and varying in the microbes present between individuals, with different bacteria causing the same clinical manifestation [38]. Furthermore, not all microorganisms have a negative impact on health as a vast range of species including members of *Pasteurellaceae* and *Prevotellaceae* are common constituents of both a healthy and diseased oral cavity [39, 40]. Therefore, it is important to ensure that a product containing *S. salivarius* designed for the oral cavity does not negatively impact neither the natural microbiome present nor the homeostatic inflammatory environment.

The application of probiotic gum on a daily-dose regimen for seven days followed by a further 7-day washout period did not modify the microbiota profiles of the healthy volunteers, as has been shown with probiotic yogurt and the gut microbiota [41]. By day 14, there was a shift in microbiota profile of few participants; however, this shift was irrespective of whether the person was administering the probiotic or on the placebo control. This was surprising because it suggests that regular gum use may impact the salivary microbiota in a proportion of the population, which is in contrast to Takeuchi et al*.*[42] and Söderling et al*.* [43] that both demonstrated no change in microbiota with regular gum use; however, the populations studied were exclusively men and children, respectively. Narrowing the study design to these specific groups may attribute to the reason no significant observations were observed but further studies would be required.

Addition consumption of *S. salivarius* K12 in the probiotic gum group did not increase the relative proportion of *Streptococcaceae* compared to the control group. As *S. salivarius* is the predominant commensal *Streptococcus* in the oral cavity [6], it is reasonable to presume the population of *Streptococcaceae* measured was mostly *S. salivarius*. Also, the additional consumption of *S. salivarius* K12 did not alter the natural immune balance in the mouth, demonstrating the potential for this therapy to be helpful without detriment to the native environment.

*S. salivarius* is often located on the dorsal surface of the tongue, and therefore would not be in high abundance in sub-gingival sites, the location where pathogens mainly cause inflammation leading to disease. Fortunately, the effector identified in this study would be able to gain access to these sites if released into saliva.

## Conclusion

This study demonstrated that *S. salivarius* K12 and M18 were able to produce a proteinaceous small molecule capable of inhibiting IL-6 and IL-8 activation of primary human gingival fibroblasts by periodontal disease pathogens. However, this molecule was not a bacteriocin and was not capable of inhibiting the growth of these pathogens. The study also demonstrated that co-administration of the effector and pathogen is not necessary and *S. salivarius* can be applied prior to pathogen exposure. This administration does not alter the native salivary microbiota nor stimulate an immune response. This shows *S. salivarius* would warrant further study using a population predisposed to periodontal disease.

## Supplementary Information

**Additional file 1: Figure S1**. Chewing Gum Study Design. Figure S1 demonstrates an overview of the design for the chewing gum study. Participants provided samples at Baseline (Day 1), 24h (Day 2), 7 days (Day 8) and after a 7-day washout period (Day 15).

**Additional file 2: Figure S2**. Bacterial attachment to primary human gingival fibroblasts. Bacterial attachment to primary human gingival fibroblasts in vitro following 8 hours co- incubation. *S. salivarius* K12 (K12); *S. salivarius* M18 (M18); *L. reuteri* RC-14; *S. mutans* ATCC25175. Assay was carried out in triplet on three separate occasions. Samples were analysed using a one-way ANOVA with Dunnett’s multiple comparison test with K12 as the control (* p < 0.05 compared to K12 attachment). Error bars represent ± standard error of the mean. Figure S2 demonstrates the level of attachment of a common probiotic *Lactobacillus reuteri* RC-14 showing *S. salivarius* K12 and M18 have much higher levels of attachment.

**Additional file 3: Table S1.** Percent decrease of IL-6 and IL-8 cytokine response when primary gingival fibroblasts are stimulated with oral pathogens and either *S. salivarius* K12 or M18 added simultaneous or as a pre-treatment. Percent decrease calculated based on respective oral-pathogen alone.

## Data Availability

Data for the current study is available upon request to the corresponding author. Parts of this manuscript contain data present in KWM thesis, freely available at http://ir.lib.uwo.ca/etd/2816.

## Abbreviations

CBA: Columbia blood agar
CFUs: Colony forming units
MEM: Minimum essential medium
OD_600_: Optical density measure at 600 nm
OTUs: Operational taxonomic units
PBS: Phosphate-buffered saline
PCoA: Principal component analysis
