# Supplementary figures and images for: Streptococcus salivarius inhibits immune activation by periodontal disease pathogens
Source: BMC Oral Health. 2021 May 7;21:245. doi: 10.1186/s12903-021-01606-z (PMC8103598; doi:10.1186/s12903-021-01606-z)

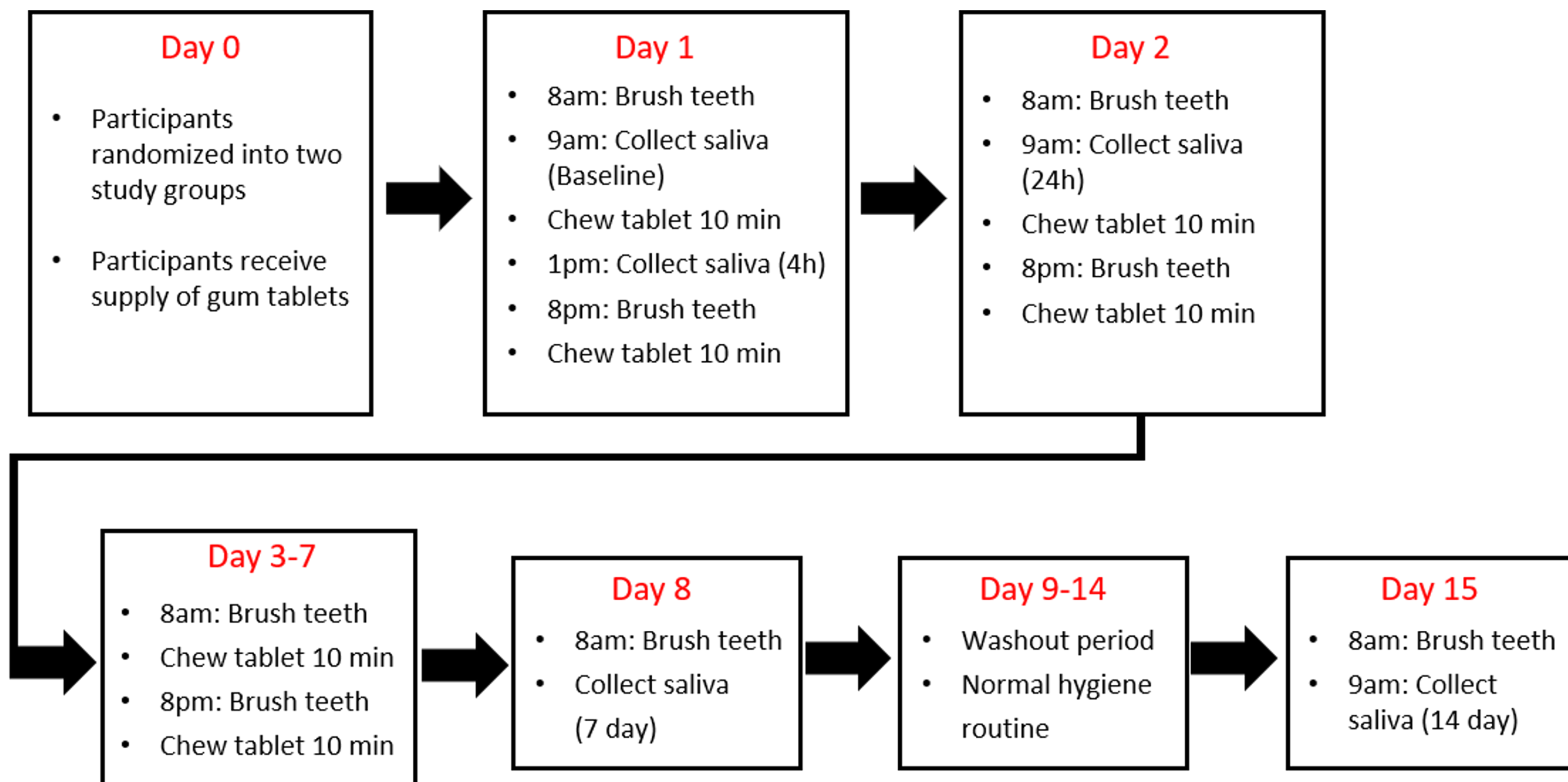

Supplement: Supplementary file 1 — Additional file 1: Figure S1. Chewing Gum Study Design. Figure S1 demonstrates an overview of the design for the chewing gum study. Participants provided samples at Baseline (Day 1), 24h (Day 2), 7 days (Day 8) and after a 7-day washout period (Day 15). [file 12903_2021_1606_MOESM1_ESM.pdf]

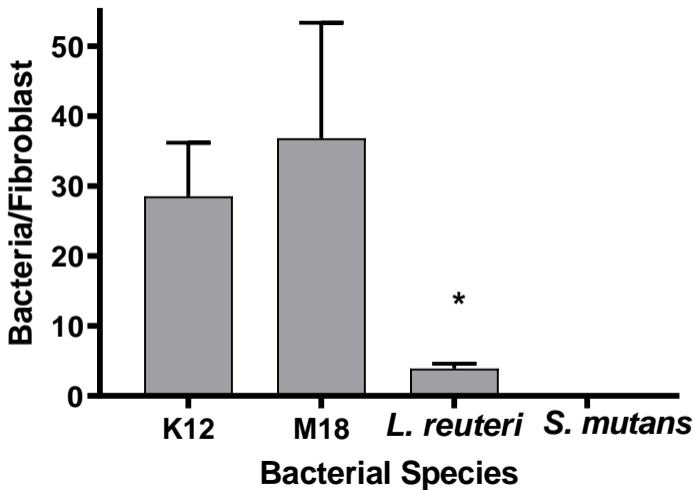

Supplement: Supplementary file 2 — Additional file 2: Figure S2. Bacterial attachment to primary human gingival fibroblasts. Bacterial attachment to primary human gingival fibroblasts in vitro following 8 hours co- incubation. S. salivarius K12 (K12); S. salivarius M18 (M18); L. reuteri RC-14; S. mutans ATCC25175. Assay was carried out in triplet on three separate occasions. Samples were analysed using a one-way ANOVA with Dunnett’s multiple comparison test with K12 as the control (* p < 0.05 compared to K12 attachment). Error bars represent ± standard error of the mean. Figure S2 demonstrates the level of attachment of a common probiotic Lactobacillus reuteri RC-14 showing S. salivarius K12 and M18 have much higher levels of attachment. [file 12903_2021_1606_MOESM2_ESM.pdf]
