# Supplementary material for: Streptococcus salivarius inhibits immune activation by periodontal disease pathogens
Source: BMC Oral Health. 2021 May 7;21:245. doi: 10.1186/s12903-021-01606-z (PMC8103598; doi:10.1186/s12903-021-01606-z)
Supplement: Supplementary file 3 — Additional file 3: Table S1. Percent decrease of IL-6 and IL-8 cytokine response when primary gingival fibroblasts are stimulated with oral pathogens and either S. salivarius K12 or M18 added simultaneous or as a pre-treatment. Percent decrease calculated based on respective oral-pathogen alone. [file 12903_2021_1606_MOESM3_ESM.docx]

**Supplementary Table 1.** Percent decrease of IL-6 and IL-8 cytokine response when primary gingival fibroblasts are stimulated with oral pathogens and administered with *S. salivarius* K12 and M18 when added simultaneous or as a pre-treatment to primary gingival fibroblasts. Percent decrease calculated based on respective oral-pathogen alone.

| Bacteria | K12^†^ | | M18^‡^ | | K12-Pretreated | | M18-Pretreated | |
| --- | --- | --- | --- | --- | --- | --- | --- | --- |
|  | **IL-6 (%)** | **IL-8 (%)** | **IL-6 (%)** | **IL-8 (%)** | **IL-6 (%)** | **IL-8 (%)** | **IL-6 (%)** | **IL-8 (%)** |
| PG^§^ | 34.1 | 72.9 | 68.8 | 88.6 | 30.0 | 63.8 | 71.1 | 88.6 |
| AA^¶^ | 48.6 | 66.8 | 43.8 | 61.5 | 45.1 | 63.2 | 52.8 | 70.8 |
| FN^•^ | 53.6 | 73.2 | 58.5 | 80.2 | 60.6 | 78.6 | 68.0 | 83.5 |
| PG+AA+FN | 64.6 | 67.2 | 74.7 | 86.8 | 60.0 | 73.7 | 80.7 | 89.5 |
| Average  (±SD | 50.2 ± 11 | 70.0 ± 3.0 | 61.5±11.7 | 79.3±10.7 | 48.9±12.6 | 69.8±6.5 | 68.1±10.0 | 83.1±7.5 |

^†^*S. salivarius* K12; ^‡^*S. salivarius* M18; ^§^*P. gingivalis*; ^¶^*A. actinomycetemcomitans*; ^•^*F. nucleatum*
